# Atomic Force Mechanobiology of Pluripotent Stem Cell-Derived Cardiomyocytes

**DOI:** 10.1371/journal.pone.0037559

**Published:** 2012-05-18

**Authors:** Jianwei Liu, Ning Sun, Marc A. Bruce, Joseph C. Wu, Manish J. Butte

**Affiliations:** 1 Department of Pediatrics, Division of Immunology & Allergy, Stanford University, Stanford, California, United States of America; 2 Department of Medicine, Division of Cardiology, Stanford University, Stanford, California, United States of America; Swiss Federal Institute of Technology Zurich, Switzerland

## Abstract

We describe a method using atomic force microscopy (AFM) to quantify the mechanobiological properties of pluripotent, stem cell-derived cardiomyocytes, including contraction force, rate, duration, and cellular elasticity. We measured beats from cardiomyocytes derived from induced pluripotent stem cells of healthy subjects and those with dilated cardiomyopathy, and from embryonic stem cell lines. We found that our AFM method could quantitate beat forces of single cells and clusters of cardiomyocytes. We demonstrate the dose-responsive, inotropic effect of norepinephrine and beta-adrenergic blockade of metoprolol. Cardiomyocytes derived from subjects with dilated cardiomyopathy showed decreased force and decreased cellular elasticity compared to controls. This AFM-based method can serve as a screening tool for the development of cardiac-active pharmacological agents, or as a platform for studying cardiomyocyte biology.

## Introduction

Diseases of cardiomyocytes, either primary (e.g., genetic cardiomyopathies) or acquired (e.g., myocardial infarction), are of major importance to health across the world [1]. Understanding the physiology and pathophysiology of these vital cells has been the subject of research for over two centuries. Obtaining human biopsy specimens from diseased patients, however, requires expensive and invasive procedures, which may be poorly tolerated by children or the critically ill. Recent breakthroughs in induced pluripotent stem cells (iPSC) [2], [3] and in genetic engineering of human embryonic stem cells (hESC) [4] have made human disease-specific cardiomyocytes available for elucidating mechanisms of specific cardiac diseases. To understand the mechanobiology of these stem cell-derived cardiomyocytes, we developed a method to measure contractile forces, beat frequencies and durations, and Young's moduli of live, beating cells.

AFM was first developed to probe nanoscale features of solid materials using its high sensitivity to intermolecular forces (∼pN) and spatial resolution (∼nm). AFM has found applications in biology to measure features of cells, such as cellular elasticity. AFM has been used to study CMs in the past [5], [6], [7], but some of these efforts required synchronizing the z-piezo of the AFM with beating of the cardiomyocytes, which created fluidic disturbances that prevented accurate measurement of contraction forces. Our method is to touch the cell gently with the AFM cantilever, then lock the *z*-piezo, which forces contractions of the cell to deflect the cantilever. Multiplying the deflection by the spring constant allows calculation of the contraction force [8].

## Results

**Figure 1:**
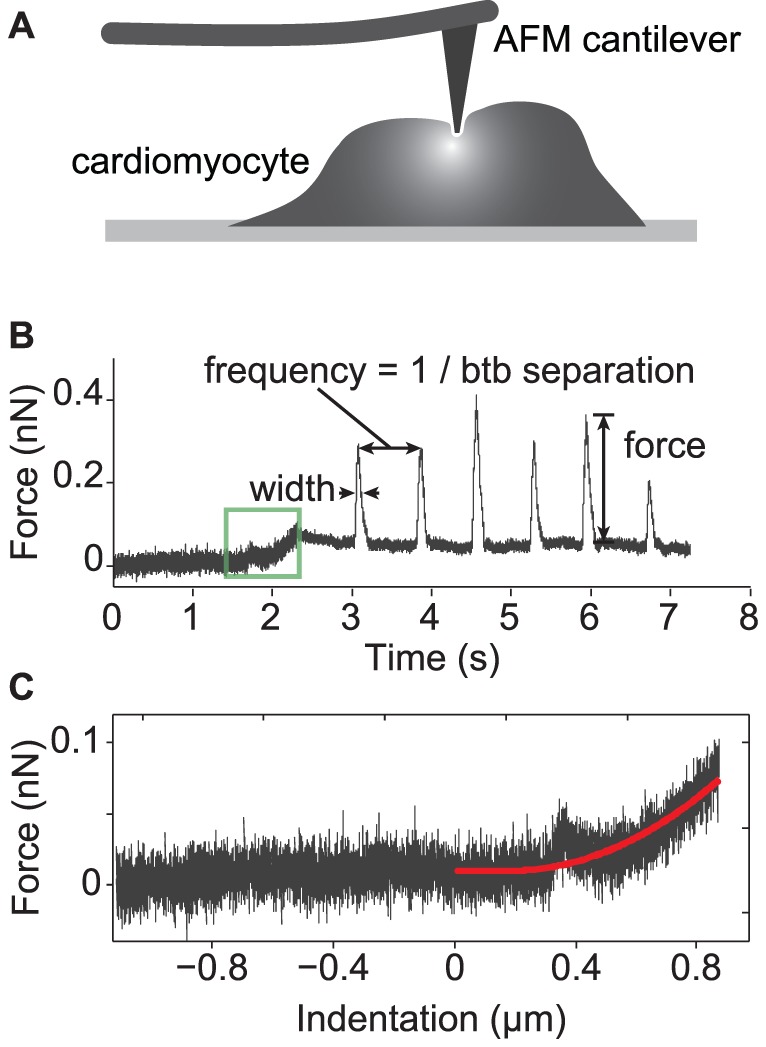
Measurement of force of CMs. (a) The AFM cantilever is brought into gentle contact with the cardiomyocyte, placing 100 pN of pre-loaded force on it. The z-piezo is locked and the cantilever tip dwells on the top of the cardiomyocyte. (b) Shows a typical force trajectory where the green box shows indentation of the cell. The contraction of the cardiomyocyte appears as peaks in the trajectory. The height, full width at half maximum (FWHM) and reciprocal of beat-to-beat separation of peaks characterize the force, duration and frequency of cardiomyocyte beat, respectively. The fit of indentation curve by using Hertz model (red curve in (c)) produces the Young's modulus of the cell membrane at the contact point.

**Figure 2:**
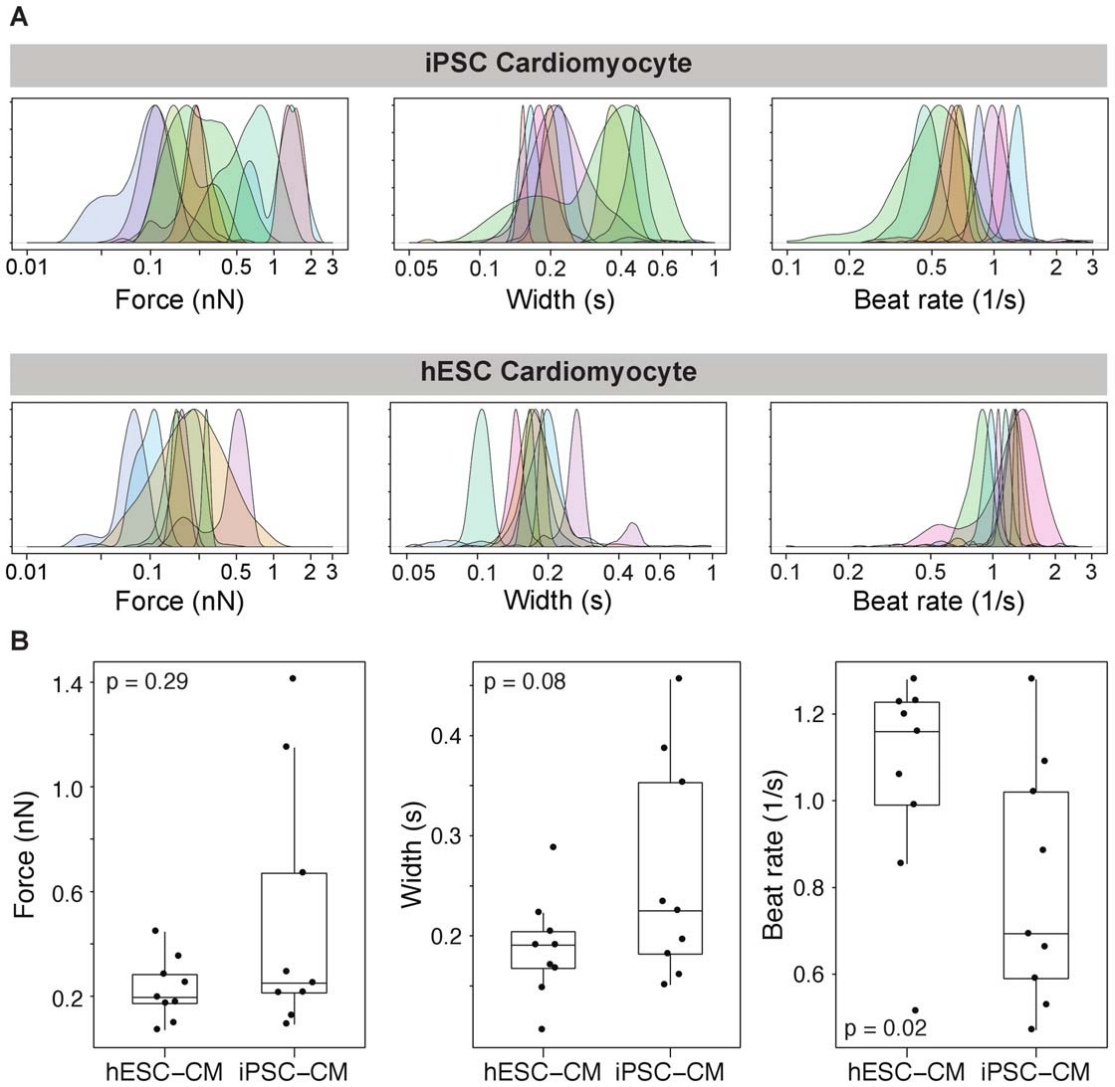
Single iPSC and hESC cardiomyocytes. Histograms of contraction force, beat width and beat rate of single iPSC-CM (a, top) and hESC-CM (a, bottom). Each curve in the plot is the smoothed histogram of the beats of a single cell measured at a single site on each cell. (b) Statistical analysis showing means of individual cells (dots), plus 25^th^, 50^th^, and 75^th^ percentile quantiles (box) and range of all points (whiskers). Statistical comparison by t test is shown.

To obtain beating cardiomyocytes from stem cells, we used either the hESC line H7 or skin fibroblast-derived iPSCs. Cardiomyocytes were successfully derived using a well-established method to differentiate these pluripotent stem cells to the cardiac lineage [9], [10]. These pluripotent stem cell-derived cardiomyocytes expressed the cardiac markers cardiac troponin T (cTnT), sarcomeric α-actinin, and myosin light chain 2a (MLC2a) (Fig. S1), though their spatial organization is more rounded than rectangular as is seen in CMs obtained from heart tissue. Moreover, they beat spontaneously *in vitro* (iPSC-CM in Video S1 and hESC-CM in Video S2). To measure the force generated by single CMs, we started by calibrating the spring constant of the AFM cantilever using the thermal noise method [8]. The typical spring constant for these cantilevers was around 0.04 N/m. The cantilever was brought into gentle contact with the surface of a CM until the cantilever registered a deflection corresponding to 100 pN of force (Fig. 1a), measuring indentation (Fig. 1c). Thereafter, we turned off feedback to the *z*-piezo and measured beats (Fig. 1b). Both iPSC- and hESC-derived cardiomyocytes contract rhythmically in the axial direction, and we noticed the force, duration and frequency vary across independent single cells (Fig. 2a). These stem-cell-derived CMs were grown on gelatin-coated, glass-bottom petri dishes, and were firmly attached – we never observed detachment of the cells due to the AFM cantilever. We found that the iPSC-derived cardiomyocytes (iPSC-CM) beat comparably to hESC-derived cardiomyocytes (hESC-CM), with contraction forces of 0.49±0.45 nN (n = 9) and 0.23±0.11 nN (n = 9), respectively (*p* = 0.29) (Fig. 2b). The total force output of these cells may be higher than we measured, because there may be lateral modes of the contraction that are not measured by this method. These measurements were done at the single point of each cell that presented the greatest beat force; we assessed the variation of beat forces at multiple points across single cells later. The mean beat rate of iPSC-CM was 0.80±0.17 beats/s (n = 9), slightly slower than that of hESC-CM at 1.06±0.23 beats/s (n = 9) (p = 0.015). The mean beat durations were 0.26±0.06 s (n = 9) and 0.19±0.05 s (n = 9) for iPSC-CMs and hESC-CMs, respectively (*p* = 0.075). Our measurements show that the CMs derived from iPSC and hESC contract with the similar mechanical properties and support the use of stem cell-derived cardiomyocytes as a model system.

**Figure 3:**
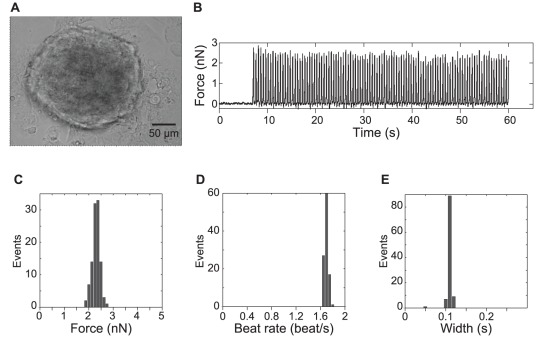
AFM measurement of iPSC-CM cluster. (a) Bright field micrograph of cluster of iPSC-CMs. (b) Contraction force trajectory. The contraction of the CM cluster shows very regular beat force (c), frequency (d) and width (e).

During culture, the iPSC-CMs can form large clusters comprising dozens of cells (Fig. 3a) that can be measured by AFM, as shown in the beat trajectory (Fig. 3b) and in the histogram of contraction force (Fig. 3c). The beating force of a single cell within the cluster was 2.37±0.16 nN (n = 106 beats). This force was stronger than the force of solitary single cells by an order of magnitude, at least partly due to the combined movement of all the cells in the cluster. The beating force of aggregated iPSC-CMs is regular (force CV = 4.8%), in contrast to isolated iPSC CMs (CV = 23%). Additionally, the aggregate contracts with uniform rhythm: 1.72±0.03 beats/s (rate CV = 1.7%) as compared to solitary iPSC-CM (CV = 20%). The consistency of contraction force and frequency shows that CMs behave more synchronously when in contact with other CMs than when solitary. This result is consistent with the known existence of cardiac gap junctions, which allow for the spread of action potentials across CMs. Together, these results show that AFM can be used to measure solitary CM and the more physiologically relevant aggregates of CM.

**Figure 4:**
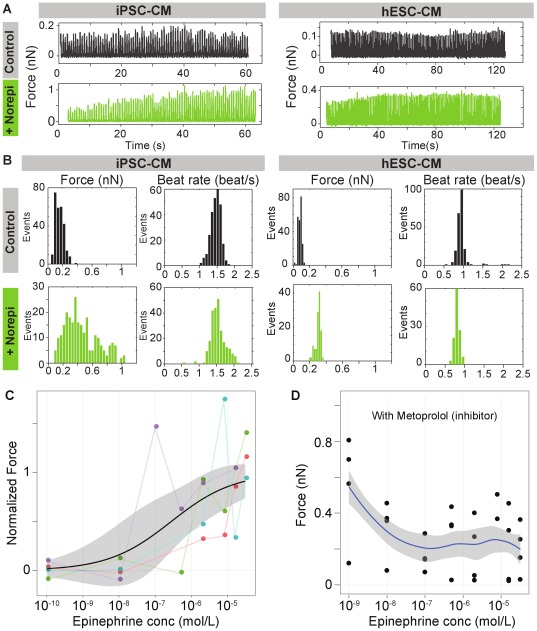
Measurement of drug effect on CMs. (a) Contraction force trajectories measured before and after treatment of iPSC- and hESC-CMs with norepinephrine. (b) Histograms of contraction force and frequency before and after treatment. (c) Beating force measured from a cluster of iPSC-CM in response to increasing doses of the adrenergic agonist epinephrine. (d) Beating force measured from a cluster of iPSC-CM in response to increasing doses of epinephrine, treated prior with the beta-blocker metoprolol.

To demonstrate the capability of AFM to measure the effect of drugs on CMs, we used norepinephrine (NE, 4-[(1*R*)-2-amino-1-hydroxyethyl]benzene-1,2-diol), a demethylated form of epinephrine that non-specifically activates both alpha-1 and beta-1 adreneregic receptors. NE has long-established effects as both a positive inotrope, and to lesser extent, a positive chronotrope as well. To study the effect of NE, we treated both solitary iPSC-CMs and hESC-CMs with NE at 100 µmol/L concentration and measured beats before treatment and immediately following treatment. As shown in the trajectories (Fig. 4a) and statistical analyses (Fig. 4b), the contraction force of iPSC-CMs increased significantly from 0.18±0.06 nN to 0.48±0.23 nN (*p*<0.001) upon treatment with NE. The drug also affected the rhythm, though the chronotropic effect was weaker than the inotropic effect. After applying NE, 21% of beats were faster than a cutoff of 1.7 beats/s as compared to 6% prior to treatment (Fig. 4b). For the hESC-CM, the contraction force increased from 0.097±0.019 nN to 0.31±0.03 nN (*p*<0.001) after treatment with NE, but there was minimal effect on the beat rate.

We next wanted to assess whether AFM could detect the more subtle effect of dose changes of an inotrope on iPSC-CM. We treated small clusters of hESC-CM with epinephrine doses from 10 nmol/L to 32 µmol/L and measured beat forces as before. We found that beat force increased as the dose increased, with an estimated EC50 of 260 nmol/L (Fig. 4c). This value is in agreement with previous reports [11]. On average, we found a 2.6-fold increase in force comparing beats prior to treatment with epinephrine and treatment with 32 µmol/L epinephrine. To test whether our AFM method could detect inhibition of adrenergic stimulation, we pre-treated hESC-CM with metoprolol (100 nmol/L) for 1 hour, then treated the CMs with epinephrine at sequentially increasing doses from 10 nmol/L to 32 µmol/L. We saw a slight decrease of beating force upon the first treatment with epinephrine (10 nmol/L dose), perhaps due to subtle movement of the tip because of fluidic shifts when the drug was introduced. Such movements were not seen with subsequent injections of epinephrine. Importantly, no increases in beat force was observed upon treatment of epinephrine in the metoprolol-treated CMs (*p* = 0.17 comparing doses of 10 nM to 32 µM epinephrine) (Fig. 4d). Together these results show that AFM can be used to measure both the inotropic and chronotropic effects of drugs and inhibitors on CMs.

**Figure 5:**
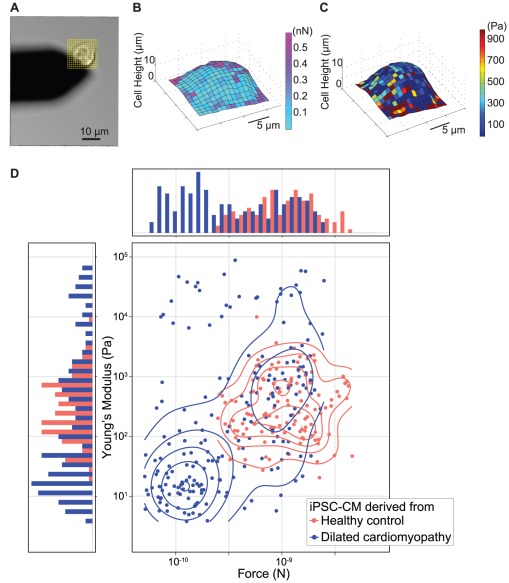
AFM dwell map of dilated cardiomyopathy iPSC-CM. (**a**) Brightfield image of an iPSC-CM showing the AFM cantilever (black shadow). The yellow grid superimposed on the cell shows the range of the dwell map. (**b**) Dwell map showing contraction forces of a single iPSC-CM at various points on the grid. (**c**) Dwell map showing Young's modulus of a single iPSC-CM at various points on the grid. The periphery of the cell had higher contraction forces and elasticity compared to the central areas. (**d**) Beating force and Young's modulus (local elasticity) measurements were obtained from dwell mapping iPSC-CM derived from either a healthy subject (red) or from a subject with dilated cardiomyopathy (DCM, blue). Single points on the plot correspond to beat force and elasticity measured at each grid points of the dwell map. Points where no contraction force was measured (e.g., on the glass slide surrounding the cell) are not shown. The contour plot (middle) represents the probability distribution of dwell mapped points in the Young's modulus vs. beating force coordinate system. The contours show that beats measured from most portions of the healthy iPSC-CM fall in a region of moderate elasticity (50 – 5 kPa) and strong force (∼1 nN), whereas some points of the dwell map of DCM iPSC-CM showed comparatively lower beat forces and lower elasticity. Corresponding histograms that flank the contour plot are the distributions of beat force (above the contour plot) and elasticity (left of the contour plot), respectively.

Because the orientation of actin-myosin filaments within a cardiomyocyte is anisotropic, different parts of the CM show different amounts of movement and contractile forces with each beat. To measure the spatial heterogeneity of contraction force, we developed a method called “dwell mapping.” By superimposing a grid on the cell, we comprehensively map the cell for elasticity by nano-indenting at each point on the grid, and for beat properties by dwelling the cantilever at each point for an interval that enables the measurement of a few beats. In practice, we sampled grids comprising 100–1000 points, most of which fell onto the cell and some of which fell onto the glass surface (Fig. 5a). Because elasticity and height at the contact point could change during the beat cycle, these changes could lead to heterogeneity in the measured forces. Dwell mapping measures the local height and local elasticity (Young's modulus) of the cell simultaneously with the local contraction forces (Fig. 5b,c).

Defects in the mechanical properties of CMs may lead to cardiomyopathies [12], [13]. Dilated cardiomyopathy (DCM) is a life-threatening genetic disorder arising from mutations of many proteins including cardiac troponin T (cTnT) [14], [15]. Cardiac troponin T binds Ca^2+^, plays a critical role in the contraction of CMs, and has been shown to be critical for heart development [16]. We showed in another work that iPSC-CMs derived from patients with DCM showed significantly decreased beat forces, but comparable rates and beat durations as iPSC-CMs derived from healthy siblings. The patient showed a typical clinical presentation of DCM [9]. We measured dwell maps of an iPSC-CM derived from a patient with DCM and found phenotypic differences compared to a healthy iPSC-CM. The contraction force histogram and Young's modulus histogram obtained from dwell maps of the DCM iPSC-CM show bimodal distributions (Fig. 5d, sides, DCM in blue). By contrast, the force histogram obtained from dwell maps of the healthy control iPSC-CM (Fig. 5d, red) shows a single population of points in terms of beating force and Young's modulus. Nonparametric bootstrap analysis of all beating points on the dwell maps gives a mean force of 1.35 nM for the control cell (95% confidence interval 1.18 nN–1.54 nN) and 0.55 nN for the DCM cell (95% CI 0.48 nN–0.64 nN)(Mann-Whitney *p*<10^−16^). Similarly, nonparametric bootstrap analysis of log-transformed Young's Moduli showed a mean elasticity of 296 Pa for the control cell (95% CI 244–367 Pa) and 167 Pa for the DCM cell (95% CI 116–235 Pa) (Mann-Whitney *p* = 0.0006). To compare the points of the two dwell maps, we used a two-dimensional (i.e., Force, Young's Modulus) Kolmogorov-Smirnov test and found the DCM and control maps are statistically distinct (*p* = 3×10^−60^). These results from dwell-mapping studies show that iPSC-CM from patients with DCM show increased populations of points of low elasticity and weak contraction. These results suggest that mutation of cTnT could both compromise filament structure and weaken contractile force.

## Discussion

The results demonstrate several uses of our AFM method to the study of cardiomyocytes. By setting the AFM probe to dwell on the cell, our method quantitatively measures its mechanical phenotypes, including the contractile force, beat rate and beat duration by avoiding fluidic disturbances that hampered previous attempts to study cardiomyocytes by AFM.

An important problem in the development of new cardiac agents is to determine whether a compound has inotropic (i.e., affecting force generation) or chronotropic (i.e., affecting rate) effects on the cardiomyocytes. Our AFM-based method quantitates these effects separately, and thus could facilitate pre-clinical studies of candidate drugs [17]. Because our approach combines measurement of cellular elasticity, beat force, and rate, it reveals additional parameters than could be seen by imaging positional changes of surface beads or by videomicroscopy of the cell edges [18], [19]. This approach could be used to analyze cells from patients with cardiomyopathy to understand the potential for gene therapy in these diseases [20]. We used dwell mapping to identify heterogeneity of the contraction and cellular elasticity of healthy and diseased iPSC-CMs, providing insight to the underlying pathophysiology of diseased cardiomyocytes. Overall, our experiments show that AFM can be applied in flexible ways to inform fundamental, applied, and clinical cardiac studies.

## Materials and Methods

### Generation and maintenance of pluripotent stem cells

All the protocols for this study were approved by the Stanford University Human Subjects Research Institutional Review Board. H7 hESC line was maintained on Matrigel-coated feeder-free culture dishes with mTESR-1 human pluripotent stem cell medium (Stemcell Technologies). Generation, maintenance, and characterization of patient-specific iPSC lines were performed as previously described [21], [22]. Briefly, fibroblasts were grown from skin biopsies taken from individual subjects and reprogrammed with lentiviral Yamanaka 4 factors (*Oct4, Sox2, Klf4*, and *c-MYC*) under feeder-free condition. Colonies with TRA-1-60^+^ staining and hESC-like morphology were picked, expanded, and established as individual iPSC lines. DCM iPSC lines were confirmed to contain the specific R173W mutation by genomic PCR and DNA sequencing. All established iPSC lines expressed the pluripotency markers Oct4, Nanog, TRA-1-81, and SSEA-4, and were positive for alkaline phosphatase.

### Cardiac lineage specification of pluripotent stem cells

H7 hESCs and iPSCs were differentiated to the CM lineage using a 3D differentiation protocol modified from Yang [10]. Briefly, embryoid bodies (EBs) were formed in basic media (StemPro34, Invitrogen, containing 2 mM glutamine, Invitrogen, 0.4 mM monothioglycerol, Sigma, 50 µg/mL ascorbic acid, Sigma, and 0.5 ng/mL BMP4, R&D Systems) by dissociating hESCs or iPSCs with Accutase (Sigma) to small clumps containing 10–20 cells on day 0. Cardiac specification of EBs was performed by adding BMP4 (10 ng/mL), human bFGF (5 ng/mL), and activin A (3 ng/mL) to the basic media on day 1–4. On day 4–8, EBs were refreshed with basic media containing human DKK1 (50 ng/mL) and human VEGF (10 ng/mL), followed by basic media containing human bFGF (5 ng/mL) and human VEGF (10 ng/mL) starting day 8. All factors were obtained from R&D Systems. Cultures were maintained in a 5% CO_2_/air environment. Spontaneous beating was observed as early as day 8 post differentiation. Beating EBs were separated by collagenase I into small beating clusters and single beating CMs for further analyses. Norepinephrine was obtained from Sigma Aldrich.

### Atomic force microscopy (AFM)

iPSC and hESC cardiomyocytes were seeded on a culture dish with a cover glass bottom (Fluorodish, World Precision Instruments, Inc.). Just before the experiments, the culture media was changed to Tyrode's solution (10 mM pH 7.4 HEPES, 140 mM NaCl, 1.8 mM CaCl_2_, 5.4 mM KCl, 1 mM MgCl_2_, 10 mM glucose) and maintained at 36°C for the entire experiment. To ensure that transient thermal effects were not affecting the cantilever deflection, the cantilever was equilibrated in the warm buffer prior to any experimental measurements until the deflection was unchanging, for at least 20 minutes. Beating cells were interrogated by using AFM (MFP-3D Bio, Asylum Research) that was mounted with a SiNi probe (BudgetSensors). Cells were gently contacted by the cantilever tip with 100 pN of force (i.e., force trigger) resulting in a typical cellular indentation of 200–500 nm. The cantilever tip remained in position with the Z-piezo feedback off for multiple, sequential, two-minute intervals while deflection data were collected at an acquisition rate of 2 kHz. Deflection data were converted to force by multiplying by the spring constant.

We measured cell beats for multiple, sequential intervals that were usually 1∼2 minutes long. Typical noise levels during these measurements were around 20 pN as shown in the force trajectory (Fig. 1b). The resulting data were analyzed in Matlab (MathWorks) to calculate the force, rate, and duration of each beat. To measure Young's modulus, as shown in Fig. 1c, we examined the indentation that occurred prior the deflection of cantilever reaching the trigger force. We fit the function of force vs. indentation distance by using the Hertz model, using code in the Asylum Research software. We used a Poisson's value of 0.5 for the cell. The fit produces the Young's modulus of the cell at the contact point. We noted some stress relaxation in elasticity measurements of live cells, because of reorganization of cytoskeletal and other components in response to local indentation – in this regard, the inverse Young's moduli reflect “dynamic compliance” rather than static. The contraction force of each beat is calculated from the peak value. The beat duration is equal to the full width at the half maximum (FWHM) and the beat rate is obtained from the reciprocal of the time interval to the next beat.

For the dwell mapping measurements, the AFM control program moves the piezo-driving stage to scan an area typically with 10∼30 lines and 10∼30 points per line. At each point, the AFM probe dwells on the cell for 10 s to measure contractions. From these measurements, the contraction force and cellular Young's modulus are calculated. Cell height is measured by the point of contact for each force curve at each point on the cell. The precision of the position of AFM tip is in several nanometers. However, since the cell is highly dynamic, the measured height is actually a temporal and spatial average of the area around the AFM tip. Contour plots were calculated automatically using the R package ggplot2 (function stat_density2d with bins = 5) [23]. Statistical tests were performed in R or in Matlab. The two-sample Kolmogorov-Smirnov test compares the distributions of the values in the two vectors comprising points of Force and Young's Moduli for the two samples (DCM and healthy CM). The null hypothesis is that the two vectors are from the same continuous distribution. The alternative hypothesis is that they are from different continuous distributions. The result H is 1 if the test rejects the null hypothesis at the 5% significance level; 0 otherwise.

## Supporting Information

Figure S1 **Cardiac markers on CM derived from stem cells.** **A.** Cardiomyocytes derived from iPSCs expressed cardiac-specific markers, including sarcomeric a-actinin, cardiac troponin T (cTnT), and myosin light chain 2a (MLC2a). DAPI fluorescence imaging shows the nucleus. Scale bars are 50 µm. hESC-CMs expressed similar cardiac-specific markers (data not shown). **B.** Higher resolution images of same markers on iPSC-CM showing myofibrillar patterns, confirming that a sarcomeric organization has developed in these cells.
(EPS)

Video S1 **Spontaneous beating of cluster of iPSC-CMs.**
(WMV)

Video S2 **Spontaneous beating of cluster of hESC-CMs.**
(WMV)
